# Neighborhood Income Inequality and Alcohol Use among Adolescents in Boston, Massachusetts

**DOI:** 10.3390/ijerph18168484

**Published:** 2021-08-11

**Authors:** Roman Pabayo, Daniel M. Cook, Gregory Farmer, Beth E. Molnar

**Affiliations:** 1 School of Public Health, University of Alberta, Edmonton, AB T6G 2R3, Canada; gfarmer@ualberta.ca; 2 School of Public Health, University of Nevada, Reno, NV 89557, USA; dmcook@unr.edu; 3 Bouvé College of Health Sciences, Northeastern University, Boston, MA 02115, USA; b.molnar@northeastern.edu

**Keywords:** income inequality, adolescents, alcohol consumption

## Abstract

Objectives: Previous research has indicated that area-level income inequality is associated with increased risk in alcohol consumption. However, few studies have been conducted among adolescents living within smaller area units, such as neighborhoods. We investigated whether neighborhood income inequality is associated with alcohol consumption among adolescents. Methods: We analyzed cross-sectional data from a sample of 1878 adolescents living in 38 neighborhoods participating in the 2008 Boston Youth Survey. Multilevel logistic regression modeling was used to determine the role of neighborhood income inequality and the odds for alcohol consumption and to determine if social cohesion and depressive symptoms were mediators. Results: In comparison to the first tertile of income inequality, or the most equal neighborhood, adolescent participants living in the second tertile (AOR = 1.20, 95% CI: 0.89, 1.61) and third tertile (AOR = 1.44, 95% CI: 1.06, 1.96) were more likely to have consumed alcohol in the last 30 days. Social cohesion and depressive symptoms were not observed to mediate this relationship. Conclusions: Findings indicate that the distribution of incomes within urban areas may be related to alcohol consumption among adolescents. To prevent alcohol consumption, public health practitioners should prioritize prevention efforts for adolescents living in neighborhoods with large gaps between rich and poor.

## 1. Background

Alcohol is the most commonly used and abused drug among youth in the United States [1]. Excessive drinking is responsible for more than 3200 deaths and approximately 119,000 emergency room visits for alcohol-related injuries among underage youth aged 12 to 21 years each year [2,3]. Findings from the 2017 Youth Risk Behavior found that among high school students, during the past 30 days, 30% drank some amount of alcohol, 14% participated in binge drinking behavior, 6% drove after consuming alcohol, and 17% rode with a driver who had been drinking alcohol [4]. In comparison, in 2018, more than half of the US adult population drank alcohol in the past 30 days, 16% reported binge drinking, and 7% reported heavy drinking [5].

Alcohol consumption among youth can have profound public health consequences. Youth who drink alcohol are more likely to experience unwanted, unplanned, and unprotected sexual activity, physical and sexual assault, a disruption of normal growth and sexual development, a higher risk for suicide and homicide, misuse of other drugs, and alcohol-related car crashes and other unintentional injuries, such as burns, falls, and drowning, among others [6,7].

Identified risk factors for underage consumption of alcohol consumption include age, gender, and race/ethnicity [8,9]. However, according to the Social Determinants of Health Framework, where people grow, work, live, and age play a role in population health. Income inequality, or the gap between high and low incomes within a residential area such as a neighborhood, city, county, state, or country is one example. Researchers have observed income inequality as a risk factor for alcohol consumption behavior in other settings. For example, using data from two nationally representative samples of adults, researchers identified that income inequality within US states was associated with increased odds for both light and heavy drinking [10]. However, another study did not identify a significant relationship between state income inequality and alcohol dependence [11]. In addition, researchers in Australia observed that increasing income inequality within Local Government Areas was associated with increasing rates of alcohol-attributable harm [12]. Few studies have been conducted among adolescents. One study conducted among adolescents living in 34 countries revealed that 11 and 13-year-olds in countries of high income inequality consumed more alcohol than youth in countries of low income inequality after adjustment for sex, family affluence, and country wealth [13]. Limited evidence exists linking income inequality within smaller residential areas, such as neighborhoods and urban centers, and alcohol use, particularly among teens.

Income inequality can adversely affect health because having a large gap between incomes of the poor and the wealthy in an area is associated with feelings of insecurity, shame, and misery among those who feel left behind. These feelings of shame and failure may lead to unhealthy coping behaviors and health conditions. For example, in a previous study, we observed that high income inequality was associated with greater depressive symptoms among girls, [14] and a greater likelihood of aggressive behaviors among boys [15]. Alcohol consumption may be a coping mechanism when feelings of shame arise when living within areas of elevated income inequality. In addition, income inequality may erode social cohesion, or the extent of connectedness and solidarity among neighbors. Reduced social cohesion has been identified as a risk factor for alcohol consumption [16].

The current study addresses the gap of having few studies of income inequality within smaller residential areas by investigating the relationship between income inequality measured at the neighborhood level and alcohol consumption in a sample of adolescents living in Boston, Massachusetts. We hypothesized that youth residing in neighborhoods with high income inequality would be more likely to participate in alcohol consumption.

## 2. Methods

We used data from the 2008 Boston Youth Survey (BYS), which is a cross-sectional survey of high school students in grades 9–12 in Boston public schools [17,18]. Of the thirty-two public high schools in Boston, 69% (*n* = 22) agreed to participate and were representative of all schools in the Boston area with respect to race/ethnicity of the students, school drop-out rates, and other socio-demographic variables [19].

A self-administered questionnaire was developed using reliable and valid scales that measure behaviors and experiences in the neighborhood. Each school selected a list of classrooms stratified by grades. A random sample of classes was selected for participation until 100–120 students were identified per school. All students in selected classrooms were invited to complete a paper-and-pencil survey during the spring of 2008 [19]. The sample size included 1878 students: a response rate of 69%. We used multiple imputation to address missing socio-demographic and behavioral data. However, students who did not provide the location of their residence were excluded. The imputed analytical sample, which included complete socio-demographic and individual-level social cohesion data, comprised of 1506/1878 (80.2%) students. We created five multiply-imputed datasets. Then, multilevel regression analyses were used to fit the pre-specified model to each of the imputed datasets. Next, we averaged the estimates to obtain estimated associations [20]. All analyses were completed using Stata 14.0. Those with missing data were more likely to be male, black, and older in age and to have immigrated to the USA within the last 4 years.

Ethical approval for this study was approved by the Alberta Research Information Services of the University of Alberta, study number Pro00112210. All methods were carried out in accordance with relevant guidelines and regulations. For the BYS, passive parental consent (i.e., upon informed consent, students’ parents were required to return a form if they did not want their child to participate) was used, and students were allowed to refuse to participate at any time before or during the survey administration. For the Boston Neighborhood Study (BNS) described below, telephone survey participants were read an informed consent document and their agreement attained before proceeding [19].

## 3. Study Variables

### 3.1. Individual-Level Characteristics

Students’ age, nativity (U.S. born, foreign born, arrived 4 years, and foreign born arrived >4 years), and race or ethnicity (white, black, Asian, Hispanic, and other) were also measured at the student level.

Student social cohesion was also measured by asking them for their perception of their neighborhood using five statements, which included: *I live in a neighborhood where people know and like each other; People in my neighborhood generally get along with each other; People in my neighborhood generally share the same beliefs about what is right and wrong; People in my neighborhood can be trusted*. Response options ranged from (1) strongly disagree to (4) strongly agree. The mean social cohesion score was 12.0 (standard deviation [SD] = 2.9), and the range was 5–20. The items showed high internal consistency (Cronbach α = 0.80). Tertile cut-offs were used to categorize social cohesion into low, moderate, and high values.

Depressive symptoms assessment: A brief adapted version of the Modified Depression Scale (MDS) was administered [21]. Students were asked to report the frequency of five symptoms such as being very sad, or feeling hopeless, in the past month. Summation scores were z-transformed, creating a continuous measure where a higher score is indicative of higher depressive symptoms. The adapted MDS has shown to be reliable (Cronbach’s α = 0.79) [21].

### 3.2. Outcomes

Alcohol consumption behavior: Three alcohol behavior questions were administered to assess underage drinking. Three outcomes were measured to determine if correlates differed. Never consumed alcohol (yes vs. no) allows researchers to identify characteristics of those who have not initiated drinking in adolescence [22]. Alcohol consumption in the past 30 days (yes vs. no) allows researchers to identify correlates of those who have initiated drinking, while alcohol consumption more than 3 times in the past 30 days (yes vs. no) will allow us to gain a better understanding of those who may excessively consume alcohol [23]. Given the age of the respondents (ages 13 to 19), each of these variables represents illegal behaviors, since the minimum drinking age in Massachusetts is 21 years of age.

Area-level covariates: In order to geocode each student’s residence to U.S. Census Tracts, investigators asked them for their nearest cross-street. Of the total sample, 85.9% (*n* = 1614) provided their locations. BYS investigators consulted with key informants from Boston neighborhoods to aggregate the 157 Boston Census Tracts (each with a population of approximately 4000) into 38 socially meaningful neighborhood clusters of tracts [24]. The details of this process are described elsewhere [19]. Then, neighborhood-level characteristics were created for this investigation.

### 3.3. Main Exposure of Interest

The main exposure of interest was income inequality within the Census Tract (CT), which was measured using the Gini coefficient. The Gini coefficient ranges from 0 (perfect equality, where every household in the CT has the exact same income) to 1.0 (perfect inequality, where households in the CT earn a wide range of incomes). In this investigation, the Gini coefficient was calculated for each Census Tract by the Boston Indicators Project (http://www.bostonindicators.org/, accessed on 1 June 2020), which was then linked to the BYS dataset. We categorized the Gini coefficient using the tertiles as threshold cutoffs. The Gini coefficient is based on the Lorenz curve, which is a cumulative frequency curve that compares the distribution of a specific variable with the uniform distribution that represents equality [25].

Using principal components analysis, a socioeconomic composite score, economic deprivation, was created for each of the 38 neighborhoods. Economic deprivation is comprised of U.S. Census indicators: proportion of residents living below the poverty level, proportion of households receiving public assistance, and proportion of families with a female head of household. The Cronbach α was 0.84, which is indicative of good internal consistency. A higher score was indicative of greater economic deprivation. Neighborhood economic deprivation was categorized into low, moderate, and high using tertiles as thresholds.

The Boston Neighborhood Survey (BNS) [19,26] was a random-digit dial telephone survey administered among adult residents (≥18 years). Respondents were randomly selected from a list-assisted sampling frame, which was stratified proportional to the population size of the 16 large neighborhoods defined by the Boston Redevelopment Authority, resulting in a sample size of 1710 adults in 2008. The purpose of the BNS was to enrich the BYS data with contextual information about neighborhood-level conditions and social processes perceived by adult residents [19,26]. For each of the 38 neighborhoods, neighborhood disorder, which is comprised of both social (i.e., presence or absence of drinking alcohol in public) and physical disorder (i.e., abandoned cars) scores were estimated. A combined score was created using these two indicators, with higher scores indicating greater neighborhood disorder. Tertiles were used to categorize neighborhoods into low, moderate, and high neighborhood disorder.

*Social Cohesion*: In addition to the student-level measurement of social cohesion, we also used the BNS to measure neighborhood social cohesion by adapting a reliable and valid questionnaire [27]. Respondents were asked if they strongly agreed, agreed, disagreed, or strongly disagreed with five statements. For example: “*People in my neighborhood can be trusted*” and “*People are willing to help their neighbors*”. A combined score was created and a greater score indicated higher social cohesion. Tertiles were used to categorize neighborhoods into low, moderate, and high neighborhood social cohesion.

*Neighborhood Danger*: Boston Police Department data were used to develop a global score for neighborhood danger in each of the 38 neighborhoods. Indicators included counts of criminal homicide, robbery, aggravated assault, burglary, larceny theft, vehicle theft, and arson. The higher the score, the greater the danger was within the neighborhood. Then, these indicators were matched to the U.S. Census Tracts and used to characterize the 38 neighborhoods. Tertiles were used to categorize danger within the neighborhood into low, moderate, and high.

## 4. Statistical Analysis

We used multilevel logistic modeling to investigate the relationship between neighborhood income inequality and alcohol consumption while adjusting for both individual and area-level characteristics. Since students were nested within CTs, which were nested within neighborhoods, a three-level multi-level model was initially considered—i.e., models with a random intercept specified for each CT and each neighborhood [28]. However, because a small amount of variation in alcohol consumption behavior was explained at the CT level (data not shown), we treated income inequality as an individual-level exposure, resulting in a two-level model (with neighborhood as the level-two unit).

We fitted the following sequence of models to investigate the association between neighborhood income inequality and the three alcohol consumption outcomes. First, we conducted an intercept-only model, which allowed us to calculate the Intraclass Correlation (ICC) and the 95% plausible value range. Both these estimates are an indication of the variability of the outcome explained at the individual and neighborhood levels. The 95% plausible value range provides an indication of the variability of likelihood of experiencing each outcome across neighborhoods. Second, we estimated the crude relationship between income inequality and each of the alcohol consumption outcomes. Third, we fitted models adding individual and neighborhood characteristics. Fourth, we added the sex × income inequality interaction term to estimate if the association between income inequality and alcohol consumption behavior differed between boys and girls. Since the interaction term was not significant, results were not presented. Finally, we added students’ perceptions of neighborhood social cohesion and individual depressive symptoms to estimate if perceptions mediated the relationship between neighborhood income inequality and alcohol consumption [29]. These mechanisms were evaluated using the Baron and Kenny [29] method of testing and comparing results from three different models: (1) income inequality and each of the alcohol consumption outcomes, while controlling for social cohesion and depressive symptoms; (2) income inequality and social cohesion and depressive symptoms; and (3) social cohesion and depressive symptoms and each of the alcohol consumption outcomes.

## 5. Results

Characteristics of the 1506 students attending public secondary schools in the Boston area are found in Table 1. Overall, the sample had more females (55.2%), almost half were black (40.9%), and a majority was born in the United States (70.1%). In addition, 38.1%, 11.3%, and 36.0% consumed alcohol at least once in the last 30 days, consumed alcohol more than 3 times in the last month, and never consumed alcohol, respectively. The average Gini score across the Census Tracts was 0.45 (SD = 0.06; range = 0.33 = 0.65). The Gini index score of Boston is similar to the overall value for the US, which has a score of 0.47 [30].

A summary of the neighborhood characteristics can also be found in Table 1. The average economic deprivation score was 0.02 (SD = 1.01; range = −1.79 to 2.42). The average proportion of the neighborhood that was black was 37.8% (SD = 28.1; range = −1.8 to 92.5).

The overall predicted probability was 37.7%, 10.0%, and 36.0% for alcohol consumption in the past 30 days, alcohol consumption more than 3 times in the last 30 days, and never having consumed alcohol. The 95% plausible value range from the null models showed some variation across neighborhoods concerning the outcomes: consuming alcohol in the last 30 days (28.9–47.4%); consuming alcohol at least 3 times in the last 30 days (5.1–18.5%); and never having consumed alcohol (26.3–47.0%). The ICC estimates indicated that the variance at the neighborhood level for alcohol consumption in the past 30 days, alcohol consumption more than 3 times in the last 30 days, and never having consumed alcohol was 1.2%, 6.6%, and 0.9%, respectively.

When we tested the crude relationship between income inequality and the alcohol consumption outcomes, there was no significant relationship between income inequality and never having consumed alcohol and having consumed alcohol 3 or more times in the last 30 days (Table 2). Students experienced an increased likelihood for consuming alcohol in the last 30 days in the second tertile (OR = 1.13, 95% CI: 0.87, 1.48) and third tertile (OR = 1.26, 95% CI: 0.96, 1.67) of income inequality, in comparison to those students living in the first tertile; however, findings were not significant. When individual and neighborhood-level factors were included, students living in the second tertile (OR = 1.20, 95% CI: 0.89, 1.61) of income inequality and third tertile (OR = 1.44, 95% CI: 1.06, 1.96) were more likely to consume alcohol in the previous 30 days. When the analyses included individual-level social cohesion and depressive symptoms as mediators, the effect of income inequality on the likelihood for consuming alcohol in the last 30 days remained. This is an indication that social cohesion and depressive symptoms did not mediate the relationship between income inequality and consuming alcohol in the previous 30 days. 

When the analyses included individual-level social cohesion and depressive symptoms as mediators, the effect of income inequality on the likelihood for consuming alcohol in the last 30 days remained. This is an indication that social cohesion and depressive symptoms did not mediate the relationship between income inequality and consuming alcohol in the previous 30 days. Otherwise, the inclusion of social cohesion would have eliminated or abated the relationship. The bivariate analyses, which estimate whether social cohesion and depressive symptoms acted as possible mediators between income inequality and each of the alcohol consumption outcome variables, are presented in Table 3. Income inequality was not associated with social cohesion or any of the alcohol consumption outcomes. However, an increase of one standard deviation of social cohesion was associated with increased odds of never having consumed alcohol (OR = 1.20, 95% CI: 1.08, 1.34) and decreased odds of consuming alcohol in the last 30 days (OR = 0.86, 95% CI: 0.77, 0.96). An increase in standard deviation of social cohesion was associated with decreased odds for consuming alcohol 3 times or more in the previous 30 days (OR = 0.89, 95% CI:0.76, 1.05), but the estimate was not significant. Therefore, the mediation bivariate analyses indicate that the relationship between income inequality and alcohol consumption was not mediated by social cohesion in this study.

## 6. Discussion

The objectives of our study were twofold: to estimate the association between neighborhood income inequality and alcohol consumption and to estimate whether social cohesion mediated the relationship. Our multilevel analysis of individual-level data collected among adolescents attending public schools in Boston and neighborhood-level data allowed us to observe a significant association between income inequality and odds for consuming alcohol in the previous 30 days. We also found that social cohesion or depressive symptoms were not mediators but might be predictive of alcohol consumption.

Findings from this study are consistent with previous work that indicated youth [13] and adults [10,11,12] exposed to income inequality experienced an increased likelihood in daily and weekly alcohol consumption. This investigation adds to the literature by studying the effect of income inequality within neighborhoods, which are smaller residential areas and are more proximal to the individual. Thus, they may play a more influential role on health behaviors and thus overall health and well-being. Although researchers in Australia utilized an ecological cross-sectional study to identify associations between Local Government Area income inequality and rates of alcohol-attributable hospitalization and death among adults, no studies have been conducted using such smaller area units among youth.

Findings imply that income inequality is associated with alcohol consumption in the past 30 days and that this potential main effect is observed among all adolescents regardless of other sociodemographic characteristics, such as income and gender. In other words, we did not observe any heterogeneous associations across socio-demographic groups. This agrees with other social epidemiologists who argue that income inequality is equally harmful to all members of society, regardless of income. One possible explanation is that the erosion of social cohesion, or the loss of trust, between members of society is bad for all members of society, not just among members of a particular socio-demographic group.

In this investigation, we tested two potential mechanisms theorized to explain how income inequality can lead to alcohol consumption. The structural inequality hypothesis posits that greater inequality may cause weaker social bonds and thus erode social cohesion [16,31]. In this investigation, social cohesion was not observed to act as a mediator between income inequality and alcohol consumption behavior. However, greater social cohesion was protective against consuming alcohol in the previous 30 days, which is consistent with the literature. Researchers who conducted a systematic review assessing the role of community social cohesion on alcohol consumption behavior found inconsistent evidence [32]. One previous study conducted among Swedish adolescents aged 13–18 indicated that those within neighborhoods with low social cohesion experienced approximately 60% increased odds of high alcohol consumption in comparison to those living in more socially cohesive neighborhoods [33]. Similar findings were observed among adolescents in Japan [34] and the former Soviet Union [35].

The relative deprivation pathway is another potential mechanism in which income inequality may be harmful to health [16]. Greater income inequality may lead to social comparisons that can lead to feelings of frustration and shame, which in turn lead to adverse mental health outcomes such as depression [16,31]. A pooled analysis of 12 studies identified in a recent systematic review demonstrated a greater risk of depression in populations with higher income inequality in comparison to populations living with lower income inequality [36]. Four of these studies were conducted among adolescents [14,37,38,39]. In the same BYS sample, we have previously identified a relationship between neighborhood income inequality and depressive symptoms among adolescent girls but not boys [14]. Furthermore, exhibiting depressive symptoms is a risk factor for alcohol use among children and adolescents [40,41]. Therefore, the relative deprivation theory posits that greater income inequality leads to social comparisons and increased feelings of shame, and it also places youth at greater risk for developing depressive symptoms, which then leads to a greater risk for alcohol consumption. Although exhibiting depressive symptoms was not a mediator between income inequality and alcohol consumption behavior, it was a significant risk factor for consuming alcohol in the past 30 days. Thus, it remains a possibility that alcohol consumption may be a coping strategy due to the stress and shame brought on by social comparisons.

Findings from this investigation should be interpreted considering study limitations. Cross-sectional data were used to investigate the role of income inequality on the odds of alcohol consumption behavior and thus temporality could not be estimated. Residual confounding could be an issue because individual-level socio-economic covariates such as household income or parental education were not collected; thus, observed relationships may be spurious. The Baron and Kenny method [29] of mediation assessments might lead to biased results because of unmeasured confounding that may exist between the mediators and outcomes [42]. Lastly, findings might not be generalizable to populations that differ significantly in comparison to Boston.

## 7. Conclusions

Overall, findings from this study suggest that neighborhood income inequality is associated with alcohol consumption in the past 30 days among adolescents living in an American urban setting, but it was not associated with never having drunk alcohol or drinking alcohol three or more times in the last 30 days. In addition, we did not find evidence that social cohesion or depressive symptoms were pathways within this relationship. Nonetheless, by knowing where adolescents reside, we may be able to identify who is potentially at greatest risk for alcohol consumption. Alcohol use during adolescence may develop into alcohol use disorders later in adulthood [43]; thus, prevention among those most at risk is warranted. Future investigations should look longitudinally to estimate whether neighborhood income inequality is a causal factor of alcohol use among adolescents and to gain a better understanding of the mechanisms involved.

## Figures and Tables

**Table 1:** Sociodemographic characteristics of adolescents: Boston Youth Study, 2008.

Covariate	*n*	%
Gender		
Female	831	55.2
Male	675	44.8
Age, years		
14	121	8.0
15	298	19.8
16	408	27.1
17	395	26.2
18	208	13.8
19	69	4.6
Race/ethnicity		
White	146	9.7
Black	616	40.9
Asian	134	8.9
Hispanic	501	33.3
Other	109	7.2
Immigrant status		
US-born	1056	70.1
Immigrant ≤ 4 years	142	9.4
Immigrant > 4 years	308	20.5
Social cohesion		
Low	586	38.9
Moderate	433	28.8
High	487	32.3
Never consumed alcohol		
No	964	64.0
Yes	542	36.0
Consumed alcohol in the last 30 days		
No	932	61.9
Yes	574	38.1
Consumed alcohol ≥3 times in the last 30 days		
No	1336	88.7
Yes	170	11.3
Neighborhood-level charactertistics	Mean (SD)	Max, Min
Gini coefficient (census tract)	0.46 (0.07)	0.33, 0.65
Economic deprivation	0.00 (1.00)	−1.79, 2,42
Neighborhood danger	0.00 (1.00)	−1.17, 3.46
Neighborhood disorder	2.87 (0.49)	2.05, 3.98
Proportion black	37.3 (27.9)	1.77, 92.54
Social cohesion	3.6 (0.21)	3.23, 4.01

**Table 2:** The relationship between income inequality and alcohol consumption among students participating in the Boston Youth Study, 2008.

	Never Consumed Alcohol				Consumed Alcohol in the Last 30 Days				Consumed Alcohol 3 or More Times in the Last 30 Days			
	Crude	Adjusted			Crude	Adjusted			Crude	Adjusted		
	OR	OR	OR	OR	OR	OR	OR	OR	OR			
	95% CI	95% CI	95% CI	95% CI	95% CI	95% CI	95% CI	95% CI	95% CI	95% CI	95% CI	95% CI
**Neighborhood Charateristics**												
**Gini**												
T1 (ref)	1.00				1.00				1.00			
T2	0.95	0.90	0.88	0.89	1.13	1.20	1.22	1.21	1.07	1.13	1.14	1.13
	0.74, 1.22	0.68, 1.21	0.65, 1.19	0.66, 1.20	0.87, 1.48	0.89, 1.61	0.91, 1.64	0.90, 1.64	0.67, 1.72	0.72, 1.77	0.73, 1.79	0.72, 1.78
T3	0.87	0.78	0.77	0.79	1.26	1.44	1.45	1.43	1.00	1.19	1.19	1.16
	0.67, 1.13	0.57, 1.06	0.57, 1.06	0.57, 1.08	0.96, 1.67	1.06, 1.96	1.07, 1.97	1.05, 1.95	0.61, 1.65	0.73, 1.92	0.74, 1.93	0.71, 1.89
**Economic Deprivation**												
Low (ref)		1.00				1.00				1.00		
Moderate		1.32	1.34	1.24		0.63	0.62	0.65		0.73	0.73	0.76
		0.87, 1.98	0.89, 2.02	0.81, 1.88		0.41, 0.95	0.41, 0.94	0.43, 1.00		0.39, 1.38	0.39, 1.38	0.40, 1.45
High		1.49	1.53	1.37		0.59	0.57	0.63		0.44	0.44	0.47
		0.91, 2.43	0.94, 2.51	0.83, 2.26		0.36, 0.96	0.35, 0.94	0.38, 1.03		0.20, 0.96	0.20, 0.95	0.21, 1.01
**Neighborhood Danger**												
Low (ref)		1.00				1.00				1.00		
		0.96	0.99	1.03		1.10	1.06	1.04		1.56	1.53	1.48
Moderate		0.65, 1.42	0.67, 1.47	0.69, 1.53		0.75, 1.62	0.72, 1.57	0.70, 1.53		0.84, 2.88	0.83, 2.84	0.80, 2.75
		0.69	0.72	0.67		1.38	1.32	1.41		1.96	1.91	1.99
High		0.49, 0.98	0.51, 1.03	0.47, 0.96		0.98, 1.96	0.93, 1.88	0.99, 2.01		1.12, 3.44	1.09, 3.36	1.13, 3.51
**Neighborhood Disorder**		1.00				1.00				1.00		
Low (ref)		0.99	0.99	0.97		1.29	1.29	1.31		1.93	1.92	1.48
Medium		0.67, 1.46	0.67, 1.47	0.65, 1.45		0.88, 1.90	0.88, 1.90	0.89, 1.94		1.07, 3.47	1.06, 3.45	0.80, 2.75
High		1.08	0.99	1.02		1.07	1.07	1.10		1.12	1.11	1.15
		0.67, 1.72	0.67, 1.72	0.63, 1.65		0.67, 1.70	0.67, 1.70	0.69, 1.77		0.53, 2.34	0.53, 2.33	0.55, 2.40
**Proportion Black**		1.00				1.00				1.00		
Low (ref)		0.65	0.66	0.64		1.19	1.17	1.19		1.79	1.78	1.79
Medium		0.45, 0.93	0.46, 0.95	0.44, 0.92		0.83, 1.70	0.81, 1.67	0.82, 1.70		1.02, 3.14	1.02, 3.12	1.02, 3.14
		0.80	0.82	0.83		0.97	0.95	0.94		2.85	2.81	2.79
High		0.50, 1.30	0.51, 1.33	0.51, 1.35		0.60, 1.56	0.59, 1.53	0.58, 1.52		1.33, 6.08	1.32, 6.01	1.30, 5.99
**Social Cohesion**												
Low (ref)		1.00				1.00				1.00		
		0.98	0.99	1.00		0.79	0.78	0.76		1.36	1.36	1.32
Medium		0.67, 1.44	0.67, 1.46	0.68, 1.48		0.53, 1.16	0.53, 1.15	0.51, 1.12		0.71, 2.63	0.70, 2.61	0.69, 2.56
		1.15	1.12	1.09		0.74	0.76	0.76		1.71	1.73	1.77
High		0.69, 1.92	0.67, 1.88	0.64, 1.83		0.44, 1.23	0.46, 1.26	0.46, 1.28		0.71, 4.15	0.71, 4.19	0.73, 4.30
**Individual Characteristics**												
**Gender**												
Male (ref)		1.00				1.00				1.00		
Female		0.85	0.85	1.01		0.95	0.95	0.81		0.71	0.71	0.61
		0.68, 1.06	0.68, 1.06	0.80, 1.27		0.77, 1.18	0.77, 1.18	0.65, 1.01		0.51, 0.99	0.51, 0.99	0.43, 0.86
**Age in Years**												
14 (ref)		1.00				1.00				1.00		
15		0.71	0.72	0.75		1.24	1.22	1.18		1.18	1.18	1.13
		0.46, 1.09	0.47, 1.11	0.48, 1.15		0.79, 1.95	0.78, 1.93	0.74, 1.86		0.52, 2.68	0.52, 2.67	0.50, 2.58
16		0.49	0.49	0.53		1.36	1.35	1.26		2.28	2.28	2.16
		0.33, 0.75	0.32, 0.75	0.34, 0.80		0.88, 2.10	0.87, 2.09	0.81, 1.96		1.07, 4.87	1.07, 4.85	1.01, 4.62
17		0.46	0.46	0.47		1.58	1.60	1.52		1.95	1.96	1.90
		0.30, 0.70	0.30, 0.69	0.31, 0.73		1.02, 2.45	1.03, 2.48	0.97, 2.36		0.91, 4.19	0.91, 4.22	0.88, 4.09
18		0.36	0.36	0.38		2.05	2.04	1.90		2.52	2.53	2.35
		0.22, 0.58	0.22, 0.58	0.24, 0.62		1.27, 3.30	1.26, 3.30	1.17, 3.09		1.12, 5.71	1.12, 5.72	1.03, 5.35
19		0.28	0.29	0.31		3.25	3.15	2.94		4.54	4.45	4.18
		0.15, 0.55	0.15, 0.56	0.16, 0.61		1.71, 6.18	1.65, 5.99	1.53, 5.64		1.76, 11.73	1.72.11.51	1.60, 5.35
**Race/Ethnicity**												
White (ref)		1.00				1.00				1.00		
		1.32	1.40	1.35		0.70	0.66	0.69		0.22	0.21	0.21
Black		0.85, 2.04	0.90, 2.18	0.87, 2.11		0.46, 1.07	0.43, 1.01	0.45, 1.06		0.12, 0.40	0.12, 0.39	0.12, 0.39
		2.87	2.92	3.05		0.31	0.31	0.30		0.15	0.15	0.15
Asian		1.71, 4.81	1.74, 4.92	1.80, 5.17		0.18, 0.55	0.18, 0.54	0.17, 0.53		0.06, 0.37	0.06, 0.37	0.06, 0.36
		0.80	0.88	0.83		1.10	1.01	1.07		0.55	0.53	0.53
Hispanic		0.52, 1.22	0.57, 1.35	0.54, 1.28		0.74, 1.65	0.67, 1.52	0.71, 1.61		0.32, 0.92	0.31, 0.90	0.31, 0.90
		0.96	1.03	1.04		0.69	0.65	0.63		0.47	0.46	0.40
Other		0.54, 1.69	0.58, 1.82	0.59, 1.86		0.40, 1.19	0.37, 1.12	0.36, 1.11		0.22, 1.00	0.22, 0.98	0.20, 0.92
**Immigrant Status**												
US Born (ref)		1.00				1.00				1.00		
		1.87	1.91	1.81		0.56	0.55	0.58		0.86	0.85	0.90
Immigrant ≤ 4 years		1.27, 2.74	1.30, 2.81	1.22, 2.67		0.37, 0.86	0.36, 0.84	0.38, 0.89		0.44, 1.67	0.43, 1.65	0.46, 1.76
		1.07	1.08	1.06		1.06	1.05	1.06		1.25	1.24	1.28
Immigrant > 4 years		0.81, 1.41	0.82, 1.42	0.80, 1.41		0.81, 1.39	0.80, 1.37	0.81, 1.40		0.83, 1.88	0.82, 1.87	0.85, 1.93
**Social Cohesion**												
Low (ref)		1.00			1.00	1.00			1.00	1.00		
Medium			1.15				0.85				0.89	
			0.87, 1.51				0.65, 1.11				0.59, 1.34	
High			1.50				0.68				0.83	
			1.15, 1.97				0.52, 0.90				0.55, 1.27	
**Depressive Symptons**				0.66				1.45				1.39
Z-Score				0.59, 0.75				1.29, 1.63				1.17, 1.65

**Table 3:** Bivariate analysis of the relationships between social cohesion and depressive symptoms, as potential mediators, and income inequality and alcohol consumption among boys and girls: 2008 Boston Youth Survey.

	Social Cohesion *β* (95% CI)	Depressive Symptoms *β* (95% CI)	Never Consumed Alcohol OR (95% CI)	Consumed Alcohol in the Last 30 Days OR (95% CI)	Consumed Alcohol 3 Times or More in the Last 30 Days (OR 95% CI)
Gini Tertile					
Gini T 1	ref	ref			
Gini T 2	0.02 (−0.12, 0.17)	−0.10 (−0.22, 0.02)	0.96 (0.74, 1.23)	1.13 (0.86, 1.48)	1.07 (0.68, 1.69)
Gini T 3	−0.07 (−0.23, 0.08)	−0.01 (−0.14, 0.11)	0.90 (0.69, 1.17)	1.24 (0.93, 1.64)	0.99 (0.61, 1.61)
Social Cohesion			1.20 (1.08, 1.34)	0.86 (0.77, 0.96)	0.89 (0.76, 1.05)
Depressive Symptoms			0.67 (0.59, 0.75)	1.41 (1.26, 1.58)	1.34 (1.14, 1.58)

## Data Availability

The data that support the findings of this study are available from the Harvard Youth Violence Prevention Center, but restrictions apply to the availability of these data, which were used under license for the current study, and so are not publicly available. However, data are available from the authors upon reasonable request and with permission of the Harvard Youth Violence Prevention Center. Please contact Deborah Azrael: azrael@hsph.harvard.edu.

## References

[1] U.S. Department of Health and Human Services (HHS), Office of the Surgeon General (2016). Facing Addiction in America: The Surgeon General’s Report on Alcohol, Drugs, and Health.

[2] Sacks J.J., Gonzales K.R., Bouchery E.E., Tomedi L.E., Brewer R.D. (2015). 2010 National and State Costs of Excessive Alcohol Consumption. Am. J. Prev. Med..

[3] Naeger S. (2017). Emergency Department Visits Involving Underage Alcohol Use: 2010 to 2013.

[4] Kann L., McManus T., Harris W.A., Shanklin S.L., Flint K.H., Queen B., Lowry R., Chyen D., Whittle L., Thornton J. (2018). Youth Risk Behavior Surveillance—United States, 2017. MMWR Surveill. Summ..

[5] Centers for Disease Control and Prevention Alcohol and Public Health. https://www.cdc.gov/alcohol/data-stats.htm%20-%20:~:text=According%20to%20the%20Behavioral%20Risk,and%207%25%20reported%20heavy%20drinking.

[6] Esser M.B., Guy G.P., Zhang K., Brewer R.D. (2019). Binge Drinking and Prescription Opioid Misuse in the U.S., 2012–2014. Am. J. Prev. Med..

[7] Miller J.W., Naimi T.S., Brewer R.D., Jones S.E. (2007). Binge drinking and associated health risk behaviors among high school students. Pediatrics.

[8] Patrick M.E., Schulenberg J.E. (2010). Alcohol use and heavy episodic drinking prevalence and predictors among national samples of American eighth- and tenth-grade students. J. Stud. Alcohol Drugs.

[9] Patrick M.E., Schulenberg J.E. (2013). Prevalence and predictors of adolescent alcohol use and binge drinking in the United States. Alcohol Res..

[10] Karriker-Jaffe K.J., Roberts S.C., Bond J. (2013). Income inequality, alcohol use, and alcohol-related problems. Am. J. Public Health.

[11] Henderson C., Liu X., Diez Roux A.V., Link B.G., Hasin D. (2004). The effects of US state income inequality and alcohol policies on symptoms of depression and alcohol dependence. Soc. Sci. Med..

[12] Dietze P.M., Jolley D.J., Chikritzhs T.N., Clemens S., Catalano P., Stockwell T. (2009). Income inequality and alcohol attributable harm in Australia. BMC Public Health.

[13] Elgar F.J., Roberts C., Parry-Langdon N., Boyce W. (2005). Income inequality and alcohol use: A multilevel analysis of drinking and drunkenness in adolescents in 34 countries. Eur. J. Public Health.

[14] Pabayo R., Dunn E.C., Gilman S.E., Kawachi I., Molnar B.E. (2016). Income inequality within urban settings and depressive symptoms among adolescents. J. Epidemiol. Community Health.

[15] Pabayo R., Molnar B.E., Kawachi I. (2014). The role of neighborhood income inequality in adolescent aggression and violence. J. Adolesc. Health.

[16] Kawachi I., Kennedy B.P. (1999). Income inequality and health: Pathways and mechanisms. Health Serv. Res..

[17] Rothman E.F., Johnson R.M., Azrael D., Hall D.M., Weinberg J. (2010). Perpetration of physical assault against dating partners, peers, and siblings among a locally representative sample of high school students in Boston, Massachusetts. Arch. Pediatr. Adolesc. Med..

[18] Hemenway D., Barber C.W., Gallagher S.S., Azrael D.R. (2009). Creating a National Violent Death Reporting System: A successful beginning. Am. J. Prev. Med..

[19] Azrael D., Johnson R.M., Molnar B.E. (2009). Creating a youth violence data violence data system for Boston, Massachusetts. Aust. N. Z. J. Criminol..

[20] Sterne J.A., White I.R., Carlin J.B., Spratt M., Royston P., Kenward M.G., Wood A.M., Carpenter J.R. (2009). Multiple imputation for missing data in epidemiological and clinical research: Potential and pitfalls. BMJ.

[21] Dunn E.C., Johnson R.M., Green J.G. (2012). The Modified Depression Scale (MDS): A Brief, No-Cost Assessment Tool to Estimate the Level of Depressive Symptoms in Students and Schools. School Ment. Health.

[22] Fisher L.B., Miles I.W., Austin S.B., Camargo C.A., Colditz G.A. (2007). Predictors of initiation of alcohol use among US adolescents: Findings from a prospective cohort study. Arch. Pediatr. Adolesc. Med..

[23] Ryan S.A., Kokotailo P., Committee on Substance Use and Prevention (2019). Alcohol Use by Youth. Pediatrics.

[24] O’Campo P. (2003). Invited commentary: Advancing theory and methods for multilevel models of residential neighborhoods and health. Am. J. Epidemiol..

[25] Kennedy B.P., Kawachi I., Prothrow-Stith D. (1996). Income distribution and mortality: Cross sectional ecological study of the Robin Hood index in the United States. BMJ.

[26] Rothman E.F., Johnson R.M., Young R., Weinberg J., Azrael D., Molnar B.E. (2011). Neighborhood-level factors associated with physical dating violence perpetration: Results of a representative survey conducted in Boston, MA. J. Urban Health.

[27] Sampson R.J., Raudenbush S.W., Earls F. (1997). Neighborhoods and violent crime: A multilevel study of collective efficacy. Science.

[28] Diez-Roux A.V. (2000). Multilevel analysis in public health research. Annu. Rev. Public Health.

[29] Baron R.M., Kenny D.A. (1986). The moderator-mediator variable distinction in social psychological research: Conceptual, strategic, and statistical considerations. J. Personal. Soc. Psychol..

[30] Weinberg D.H. (2011). US Neighborhood Income Inequality in the 2005–2009 Period.

[31] Harling G., Subramanian S.V., Barnighausen T., Kawachi I. (2014). Income inequality and sexually transmitted in the United States: Who bears the burden?. Soc. Sci. Med..

[32] McPherson K.E., Kerr S., Morgan A., McGee E., Cheater F.M., McLean J., Egan J. (2013). The association between family and community social capital and health risk behaviours in young people: An integrative review. BMC Public Health.

[33] Aslund C., Nilsson K.W. (2013). Social capital in relation to alcohol consumption, smoking, and illicit drug use among adolescents: A cross-sectional study in Sweden. Int. J. Equity Health.

[34] Takakura M. (2011). Does social trust at school affect students’ smoking and drinking behavior in Japan?. Soc. Sci. Med..

[35] Murphy A., Roberts B., Kenward M.G., De Stavola B.L., Stickley A., McKee M. (2014). Using multi-level data to estimate the effect of social capital on hazardous alcohol consumption in the former Soviet Union. Eur. J. Public Health.

[36] Patel V., Burns J.K., Dhingra M., Tarver L., Kohrt B.A., Lund C. (2018). Income inequality and depression: A systematic review and meta-analysis of the association and a scoping review of mechanisms. World Psychiatry.

[37] Vilhjalmsdottir A., Gardarsdottir R.B., Bernburg J.G., Sigfusdottir I.D. (2016). Neighborhood income inequality, social capital and emotional distress among adolescents: A population-based study. J. Adolesc..

[38] Goodman E., Huang B., Wade T.J., Kahn R.S. (2003). A multilevel analysis of the relation of socioeconomic status to adolescent depressive symptoms: Does school context matter?. J. Pediatr..

[39] McLaughlin K.A., Costello E.J., Leblanc W., Sampson N.A., Kessler R.C. (2012). Socioeconomic status and adolescent mental disorders. Am. J. Public Health.

[40] Johannessen E.L., Andersson H.W., Bjorngaard J.H., Pape K. (2017). Anxiety and depression symptoms and alcohol use among adolescents—A cross sectional study of Norwegian secondary school students. BMC Public Health.

[41] Wu P., Bird H.R., Liu X., Fan B., Fuller C., Shen S., Duarte C.S., Canino G.J. (2006). Childhood depressive symptoms and early onset of alcohol use. Pediatrics.

[42] Kaufman J.S., Maclehose R.F., Kaufman S. (2004). A further critique of the analytic strategy of adjusting for covariates to identify biologic mediation. Epidemiol. Perspect. Innov..

[43] Yuen W.S., Chan G., Bruno R., Clare P., Mattick R., Aiken A., Boland V., McBride N., McCambridge J., Slade T. (2020). Adolescent Alcohol Use Trajectories: Risk Factors and Adult Outcomes. Pediatrics.

